# Primary Care Continuity and Utilization Patterns for Veterans With Homeless Experience

**DOI:** 10.1001/jamanetworkopen.2025.57754

**Published:** 2026-02-02

**Authors:** Kevin R. Riggs, Aerin J. deRussy, April E. Hoge, Audrey L. Jones, Erin F. Shufflebarger, Joshua S. Richman, Ann Elizabeth Montgomery, Lillian Gelberg, Allyson L. Varley, Adam J. Gordon, Stefan G. Kertesz

**Affiliations:** 1 Birmingham VA Health Care System, Birmingham, Alabama; 2 University of Alabama at Birmingham Heersink School of Medicine; 3 Informatics, Decision-Enhancement and Analytic Sciences (IDEAS) Center, VA Salt Lake City Health Care System, Salt Lake City, Utah; 4 Program for Addiction Research, Clinical Care, Knowledge and Advocacy (PARCKA), Division of Epidemiology, Department of Internal Medicine, University of Utah School of Medicine, Salt Lake City; 5 University of Alabama at Birmingham School of Public Health; 6 VA Greater Los Angeles Health Care System, Los Angeles, California; 7 David Geffen School of Medicine at UCLA, Los Angeles, California; 8 UCLA Fielding School of Public Health, Los Angeles, California; 9 Heart Rhythm Clinical and Research Solutions, LLC, Birmingham, Alabama

## Abstract

**Importance:**

Continuity of care is a key aspect of high-quality primary care. Vulnerable populations often experience fragmented care. Some US Department of Veterans Affairs (VA) clinics offer primary care in patient aligned care teams (PACTS) tailored for veterans with homeless experience (VHE), termed H-PACTs.

**Objectives:**

To test the hypothesis that primary care continuity would be higher for VHEs in H-PACTs than for VHEs in mainstream VA PACTs and to compare other service utilization patterns by primary care clinic type.

**Design, Setting, and Participants:**

Retrospective observational cohort study including national survey data combined with VA electronic health records data from primary care clinics at 26 VA medical centers. Participants were VHEs who completed the national survey and had 2 or more primary care visits in the 12 months before the survey. The survey was completed between April and October 2018 and data were analyzed from April 2020 to November 2025.

**Exposure:**

Enrollment in H-PACTs or mainstream PACTs.

**Main Outcomes and Measures:**

Continuity was calculated using the usual provider of care (UPC) measure, which is the proportion of primary care visits with the most frequently seen clinician. High continuity was defined as a UPC of 0.75 or higher. Multivariable regression models examined the association of H-PACT enrollment with high continuity, and other utilization measures included mental health, specialty visits, emergency department (ED) visits, and hospitalizations.

**Results:**

A total of 2271 VHEs in H-PACTs (2140 [94.2%] male; 932 [41.0%] Black, 1050 [46.2%] White, and 263 [11.6%] other; mean [SD] age, 58.1 [9.3]) and 1627 VHE in mainstream PACTs (1393 [85.6%] male; 674 [41.4%] Black, 740 [45.5%] White, and 192 [11.8%] other; mean [SD] age, 60.7 [12.1]) were included. Compared with those in mainstream PACTs, VHEs in H-PACTs had a higher mean (SD) UPC (0.81 [0.23] vs 0.77 [0.25]; χ^2^_1_ = 21.6; *P* < .001) and were more likely to achieve high continuity (1483 patients [65.3%] vs 938 [57.7%]; χ^2^_2_ = 25.0; *P* < .001). After multivariable adjustment, care in H-PACTs remained associated with high continuity (odds ratio [OR], 1.48; 95% CI, 1.33-1.66). In adjusted analyses, compared with those in mainstream PACTs, VHEs in H-PACTs had significantly more primary care visits (4.6 vs 4.0; *z *score = 5.28; *P* < .001), fewer specialty visits (6.2 vs 7.9 visits; *z* score = −4.66; *P* < .001), and were less likely to have an ED visit (OR, 0.83; 95% CI, 0.75-0.92).

**Conclusions and Relevance:**

In this study, VHEs in H-PACT clinics had higher primary care continuity with no indication of substitution of specialty or emergency visits for primary care. The H-PACT model is associated with less intensive health care delivery.

## Key Points

**Question:**

How do continuity and patterns of care differ between primary care clinics tailored for veterans with homeless experience (VHEs) compared with nontailored clinics?

**Findings:**

This cohort study involving 2271 VHEs found that VHEs in tailored clinics had significantly higher continuity than VHEs in nontailored clinics, with no indication of substitution of specialty or emergency visits for primary care.

**Meaning:**

These findings suggest that tailored primary care clinics may result in higher continuity and overall less intensive health care delivery for this vulnerable population.

## Teaser

This cohort study examines whether veterans with homeless experience receiving care in homeless patient aligned care teams vs mainstream patient aligned care teams have different levels of primary care continuity.

## Introduction

Continuity is a key aspect of high-quality medical care, particularly for primary care. The Institute of Medicine has defined continuity as “care over time by a single clinician or team of health care professionals.”^1^ While results have been mixed,^2,3,4^ higher continuity has been associated with positive health outcomes, including reduced costs,^5^ less overuse of low-value care,^4,6^ and reduced emergency department (ED) visits^3^ and hospitalizations.^5,7^ Measures of continuity are beginning to be used as a health care quality indicator.^8^

Persons who are currently or recently homeless (homeless-experienced) face challenges accessing and sustaining health care due to financial barriers, lack of transportation, and stigma, which can result in fragmented or low-continuity care. The US Department of Veterans Affairs (VA) has long used a medical home model for primary care, termed patient-aligned care teams (PACTs), and began tailoring some outpatient clinics specifically for veterans with homeless experience (VHE) in 2012,^9^ termed Homeless-PACTs (H-PACTs). While there is imperfect consensus as to what tailoring involves, common features of H-PACTs include specialized staff training, smaller patient panels allowing for longer visits, integrated addiction services, and tangible assistance, like food and clothing.^10^ The goals of tailored H-PACTs were to uphold the longitudinal, comprehensive, relationship-based goals of primary care for a vulnerable population for whom those goals are not always met in conventional outpatient clinics. Several reports indicate that tailored care models draw superior care ratings from patients,^11,12,13^ but how they affect longitudinal service delivery is less clear.^14^

Maximizing continuity in primary care is important, though that objective sits in potential tension with other important goals, such as maximizing access.^15^ For example, a primary care clinic could elect to maximize access by offering same-day walk-in visits with a clinician other than a patient’s usual practitioner, and in so doing reduce continuity. Yet the same-day visit approach could prevent an urgent care or ED visit, producing similar or better outcomes at lower costs and a better patient experience. Therefore, examining continuity in isolation may not capture the full scope of health care service delivery, particularly the potential substitution of primary care visits for higher-level care (ie, specialty care or ED visits).

The present study seeks to compare both primary care continuity and other health service utilization for VHEs using VA’s H-PACTs compared with those using mainstream PACTs. We hypothesized primary care continuity would be higher for VHEs in H-PACTs, without concomitantly higher utilization of other health services, such as specialty care or ED visits.

## Methods

### Study Design and Population

This study is a secondary analysis of a national survey of VHEs that focused on patient ratings of primary care experience and was combined with additional data elements derived from VA electronic health records. The survey was completed between April and October 2018 and included VHEs (based on VA records indicating receipt of services or diagnostic codes that indicated a history of homelessness) who were engaged in primary care at the 26 VA Medical Centers (VAMCs) that operated H-PACTs at the time, with a total of 5766 respondents (response rate, 40.2%). A detailed description of the survey methodology is published elsewhere.^16^ In brief, veterans were eligible for inclusion if they had VA records indicative of homelessness in the 30 months prior to November 2017 based on diagnostic codes. Sampling was limited to VHEs at VAMCs operating the 29 largest H-PACTs at the time. Survey recruitment by mail and telephone was led by Strategic Research Group of Columbus, Ohio, with $10 remuneration to participants who provided informed consent. The electronic health records data were derived from the VA’s Corporate Data Warehouse (CDW). This study was approved by the VA’s Central institutional review board and followed Strengthening the Reporting of Observational Studies in Epidemiology (STROBE) reporting guidelines.

### Measures

The primary exposure variable was enrollment in either an H-PACT or a mainstream PACT, which was identified from VA administrative records. Enrollment status for each participant was determined by query of CDW on December 17, 2017. Each primary care site was also categorized as being located at a community-based outpatient clinic (CBOC) or located at the main VAMC. In the parent survey study, VHEs enrolled in H-PACTs were oversampled to power organizational analyses unrelated to the present study.^17^ The ratio of VHEs from H-PACTs to mainstream PACTs was approximately 2:1.

Health care utilization, including primary care visits, specialty care visits, ED visits, and hospitalizations, were derived from VA CDW for the 12 months before survey completion. For outpatient visits, we identified types of visits from 3-digit clinic location codes called stop codes. For primary care visits, we counted visits with a primary care stop code, including comprehensive women’s primary care, geriatric primary care, and general internal medicine. We included visits categorized as in-person as well as telehealth. We limited primary care visits to those where a physician or advanced practice clinician was assigned as the primary clinician for that visit (rather than a registered nurse or other PACT member). Other types of visits, such as mental health and specialty visits, were identified using VA stop codes without reference to the mode of delivery or clinician type. We categorized these other types of visits using definitions from Ferguson, et al,^18^ including homeless services (such as social work visits for shelter or housing intakes), mental health services, specialty care, rehabilitation care, ancillary care, and ED visits (all provided in VA facilities and excluding outside care paid for by VA). We used a day-count for each of the previously mentioned categories, meaning that multiple stop codes identified with the same service type on the same day were counted as a single visit. We also counted the number of inpatient admissions, and identified admissions for mental health or substance use reasons based on principal diagnosis. A list of all stop codes used is shown in eTable 1 in Supplement 1.

The measure of continuity used in this study was a form of the usual provider of care (UPC).^19,20^ The UPC is a measure of visit density—a measure of visits with a usual practitioner as a proportion of total visits (ranging from 0 to 1),^19,20^ although the numerator and denominator of the UPC are operationalized differently across studies. In some studies, the usual practitioner (numerator) is identified as an assigned practitioner,^21^ whereas in others it is the practitioner seen most frequently.^22^ For this study, we used the primary care practitioner who was seen most frequently over the 12-month observation period. Likewise, calculation of the denominator varies across studies,^23^ sometimes consisting of the total number of visits across all specialties,^7^ and other times using a subset of visits.^24^ Here, we limited the denominator to only primary care visits for 2 reasons. First, we sought to focus on the degree of continuity specifically within primary care, comparing clinic models (H-PACT vs mainstream). Second, because of the way that VA measures utilization, we were more confident in accurately identifying visits with primary care practitioners (as described previously) than in all of the various specialties, given nuances in coding encounters that vary across specialties.

When calculating UPC, participants with 0 primary care visits have an undefined continuity measure, and those with 1 primary care visit universally have perfect continuity (UPC = 1). To mitigate bias from presuming perfect continuity based on 1 visit, we excluded 1868 respondents with fewer than 2 primary care visits with a physician or advanced practice practitioner in the 12 months before the survey. We categorized primary care continuity as follows: 0.75 or above as high continuity, 0.50 to 0.74 as moderate continuity, and less than 0.50 as low continuity, similar to cut points used in other studies,^25^ and for most analyses we dichotomized a UPC of 0.75 or greater as high continuity.

Most covariates were obtained from self-report on the survey and were selected based on the Behavioral Model for Vulnerable Populations.^26^ Demographic variables included self-reported race and ethnicity (categorized as Black, White, or other, which included American Indian or Alaskan Native, Asian or Pacific Islander, and other with a free-text response option), marital status, education, and employment status. Chronic homelessness was defined as having reported 4 separate instances of homelessness in the last 3 years or affirming current homelessness with the longest episode greater than 1 year. Financial variables included difficulty affording basic needs like housing and food. Alcohol or drug problems were based on the 2-Item Conjoint Scale,^27^ which assesses having used alcohol or drugs more than you meant to and felt you wanted to or needed to cut down in the preceding year. Moderate or severe chronic pain was based on a validated 2-item screener,^28,29,30^ with current pain rated at 4 or higher on 0 to 10 scale. Psychological distress was based on summing 4 depression and anxiety items from the 4-item Patient Health Questionnaire^31^ along with 2 items assessing psychotic symptoms on the Colorado Mental Health Symptom Index (summed range 0-24; α = 0.84).^32,33^ Self-reported health was reported on a 4-point scale and dichotomized as excellent or good or fair or poor. Psychological distress was dichotomized at 10 or higher to indicate severe based on face validity: a score of 10 or higher would be attained if a person reported 5 of 6 symptoms several days a week, or if they reported 3 of 6 symptoms more than half the days a week and 1 symptom 1 or 2 days. A 6-item social support indicator was devised from 5 items from the emotional support and isolation scales of the National Institutes of Health Patient-Reported Outcomes Measurement Information Set,^34^ as well as the capacity to borrow $20. Elixhauser scores were calculated from electronic health records.

### Statistical Analysis

We first compared baseline characteristics of participants by clinic assignment (H-PACT or mainstream PACT), using the χ^2^ test for categorical variables and Wilcoxon rank-sum for continuous variables. We then compared unadjusted primary care continuity by clinic assignment (H-PACT or mainstream PACT). Because continuity scores typically decrease for those with more clinic visits, which could potentially confound a comparison if there were more visits to H-PACT vs mainstream clinics (or vice versa), we iterated this comparison after stratification by the number of clinic visits (2-3, 4-6, or ≥7). We then compared unadjusted encounter counts by clinic assignment (H-PACT or mainstream PACT).

We then examined associations with high primary care continuity using a multivariable logistic regression model adjusted for self-reported participant demographics, psychosocial indicators, medical indicators, and mental health indicators (described previously in the Measures section). The model also accounted for site as a random effect and was weighted based on the inverse probability of survey response. We used inverse probability of survey response weighting, using comparison of characteristics of respondents and nonrespondents visible through the VA’s CDW to make results more representative of the underlying population, as described elsewhere.^11^

Finally, we examined multivariable regression models to test for differences in utilization after controlling for potential confounders. We constructed linear regression models for outpatient care visit counts^35^ and negative binomial regression models for number of ED visits and hospitalizations and whether there were any hospitalizations or ED visits for the year. We used linear regression where possible so that coefficients could be interpreted as absolute additive changes in means rather than (relative) proportional changes. All models included self-reported patient demographics, psychosocial indicators, medical indicators, and mental health indicators, including site as a random effect and survey response weights. A 2-sided *P* value less than .05 was considered significant. SAS Enterprise Guide version 8.3 (SAS Institute) was used for analyses, which were completed from April 2020 to November 2025.

## Results

A total of 2271 VHEs in H-PACTs (2140 [94.2%] male; 932 [41.0%] Black, 1050 [46.2%] White, and 263 [11.6%] other; mean [SD] age, 58.1 [9.3]) and 1627 VHE in mainstream PACTs (1393 [85.6%] male; 674 [41.4%] Black, 740 [45.5%] White, and 192 [11.8%] other; mean [SD] age, 60.7 [12.1]) were included. The baseline characteristics of the final analytic cohort (3898 veterans) are shown in Table 1. VHEs in H-PACTs had slightly lower mean (SD) age than those in mainstream PACTs (58.1 [9.3] vs 60.7 [12.1] years; χ^2^_1_ = 69.4; *P* < .001), were more likely to be male (2140 patients [94.2%] vs 1393 [85.6%]; χ^2^_1_ = 82.9; *P* < .001), were more likely to have only a high school education (919 patients [41.9%] vs 536 [34.3%]; χ^2^_2_ = 23.2; *P* < .001), were more likely to have their primary care clinic in CBOCs (643 patients [28.3%] vs 334 [20.5%]; χ^2^_1_ = 30.5; *P* < .001), and were more likely to be chronically homeless (487 patients [21.4%] vs 182 [11.2%]; χ^2^_1_ = 70.2; *P* < .001). VHEs (1868 veterans) excluded for having 0 or 1 primary care visit during the study period differed from the analytic sample in few ways (eTable 2 in Supplement 1). Notably, VHEs in H-PACTs were more likely to have 0 primary care visits than those in mainstream PACTs.

**Table 1.** Baseline Participant Characteristics of Veterans Experienced in Homelessness Receiving Veterans Affairs Primary Care, Overall and by Type of Primary Care Clinic

Characteristic	Participants, No. (%)			*P* value
	Overall (N = 3898)	H-PACT (n = 2271)	Mainstream PACT (n = 1627)	
Age, y				<.001
Mean (SD)	59.2 (10.6)	58.1(9.3)	60.7 (12.1)	
<55	903 (23.2)	552 (24.3)	351 (21.6)	<.001
55-64	2031 (52.1)	1310 (57.7)	721 (44.3)	
>65	964 (24.7)	409 (18.0)	555 (34.1)	
Gender				
Female	365 (9.4)	131 (5.8)	234 (14.4)	<.001
Male	3533 (90.6)	2140 (94.2)	1393 (85.6)	
Race				
Black	1606 (41.20)	932 (41.04)	674 (41.43)	.95
White	1790 (45.92)	1050 (46.24)	740 (45.48)	
Other^a^	455 (11.67)	263 (11.58)	192 (11.80)	
Missing	47 (1.21)	26 (1.14)	21 (1.29)	
Ethnicity				
Hispanic	414 (10.62)	248 (10.92)	166 (10.20)	.27
Non-Hispanic	3384 (86.81)	1972 (86.83)	1412 (86.79)	
Missing	100 (2.57)	51 (2.25)	49 (3.01)	
Marital status				
Married	724 (18.57)	318 (14.27)	406 (25.50)	<.001
Never married	984 (25.24)	627 (28.13)	357 (22.42)	
Divorced	1841 (47.23)	1130 (50.70)	711 (44.66)	
Widowed	272 (6.98)	154 (6.91)	118 (7.41)	
Missing	77 (1.98)	42 (1.85)	35 (2.15)	
Education				
HS GED or equivalent	1455 (37.33)	919 (41.85)	536 (34.34)	<.001
More than HS GED	2302 (59.06)	1277 (58.15)	1025 (65.66)	
Missing	141 (3.62)	75 (3.30)	66 (4.06)	
Employment				
Employed	712 (18.27)	426 (19.28)	286 (18.01)	<.001
Unemployed	2079 (53.34)	1285 (58.14)	794 (50.00)	
Retired	1007 (25.83)	499 (22.58)	508 (31.99)	
Missing	100 (2.57)	61 (2.69)	39 (2.40)	
Facility type				
VAMC	2921 (74.94)	1628 (71.69)	1293 (79.47)	<.001
CBOC	977 (25.06)	643 (28.31)	334 (20.53)	
Chronic homelessness	669 (17.16)	487 (21.44)	182 (11.19)	<.001
Elixhauser score, mean (SD)	4.2 (2.4)	4.2 (2.3)	4.1 (2.4)	.20
Difficulty paying for basics				
Yes	908 (23.29)	563 (25.31)	345 (21.73)	.03
No	2904 (74.50)	1661 (74.69)	1243 (78.27)	
Missing	86 (2.21)	47 (2.07)	39 (2.40)	
Social support				
Low or poor	1415 (36.30)	856 (38.25)	559 (34.94)	.09
High or good	2423 (62.16)	1382 (61.75)	1041 (65.06)	
Missing	60 (1.54)	33 (1.45)	27 (1.66)	
Self-reported health				
Poor or fair	1881 (48.26)	1065 (48.74)	816 (52.27)	.09
Excellent or good	1865 (47.85)	1120 (51.26)	745 (47.73)	
Missing	152 (3.90)	86 (3.79)	66 (4.06)	
Self-reported alcohol problem				
Yes	1025 (26.30)	660 (29.54)	365 (22.77)	<.001
No	2812 (72.14)	1574 (70.46)	1238 (77.23)	
Missing	61 (1.56)	37 (1.63)	24 (1.48)	
Self-reported drug problem				
Yes	482 (12.37)	321 (14.37)	161 (10.04)	<.001
No	3355 (86.07)	1913 (85.63)	1442 (89.96)	
Missing	61 (1.56)	37 (1.63)	24 (1.48)	
Self-reported chronic pain				
Yes	2499 (64.11)	1449 (63.80)	1050 (64.54)	.64
No	1399 (35.89)	822 (36.20)	577 (35.46)	

Abbreviations: CBOC, community-based outpatient clinic; GED, general educational development; HS, high school; H-PACT, homeless patient aligned care team; PACT, patient aligned care team; VAMC, Veterans Affairs Medical Center.

^a^ Other includes American Indian or Alaskan Native, Asian or Pacific Islander, and Other with a free-text response option.

Unadjusted continuity comparisons are shown in Table 2. The mean (SD) UPC was higher among those in H-PACTs than those in mainstream PACTs (0.81 [0.23] vs 0.77 [0.25]; χ^2^_1_ = 21.6; *P* < .001), and those in H-PACTs were significantly more likely to have high continuity (1483 patients [65.3%] vs 938 [57.7%]; χ^2^_2_ = 25.0; *P* < .001). Those in H-PACTs were less likely to have only 2 to 3 primary care visits in the past 12 months (1059 patients [46.6%] vs 916 [56.3%]), and more likely to have 4 to 6 or 7 or more (by about 5 percentage points each). Nevertheless, those in HPACTs had consistently higher continuity across all visit count strata.

**Table 2.** Primary Care Continuity, Overall and Stratified by Number of Visits

Variable	Participants, No (%)			*P* value
	Total (N = 3898)	H-PACT (n = 2271)	Mainstream PACT (n = 1627)	
PC continuity	79.3 (23.7)	81.0 (22.7)	77.0 (24.7)	<.001
Mean (SD)				
High (0.75 to 1.00)	2421 (62.1)	1483 (65.3)	938 (57.7)	
Moderate (0.50 to <0.75)	1125 (28.9)	610 (26.9)	515 (31.7)	<.001
Low (<0.50)	352 (9.0)	178 (7.8)	174 (10.7)	
PC continuity by No. of visits				
2-3 PC visits	1975 (50.7)	1059 (46.6)	916 (56.3)	
Mean (SD) continuity	83.1 (23.3)	84.1 (23.0)	81.9 (23.6)	.03
4-6 PC visits	1290 (33.1)	797 (35.1)	493 (30.3)	
Mean (SD) continuity	77.7 (22.8)	80.5 (21.6)	73.1 (24.1)	<.001
≥7 PC visits	633 (16.2)	415 (18.3)	218 (13.4)	
Mean (SD) continuity	71.0 (23.9)	73.9 (22.7)	65.4 (25.2)	<.001

Abbreviations: H-PACT, homeless patient aligned care team; PACT, patient aligned care team; PC, primary care.

The factors associated with high primary care continuity are shown in Table 3. Being enrolled in an H-PACT was independently associated with a higher likelihood of high primary care continuity (OR, 1.48; 95% CI, 1.33-1.66; *P* < .001). Notably, receiving primary care at a clinic situated in a CBOC, compared with at a main VAMC, was also associated with higher likelihood of high primary care continuity (OR, 3.62; 95% CI, 2.64-4.97; *P* < .001).

**Table 3.** Association Between Participant Factors and High Primary Care Continuity^a^

	aOR (95% CI)	*P* value^b^
Clinic type: H-PACT vs mainstream PACT	1.48 (1.34-1.64)	<.001
Facility type: CBOC vs VAMC	3.00 (2.23-4.02)	<.001
Race (reference, White)		
Black	1.00 (0.89-1.12)	.65
Other	0.92 (0.80-1.06)	
Missing	1.08 (0.71-1.65)	
Ethnicity (reference, non-Hispanic)		
Hispanic	1.11 (0.95-1.29)	.35
Missing	0.91 (0.64-1.31)	
Marital status (reference, never married)		
Divorced	0.91 (0.81-1.01)	.07
Married	0.89 (0.77-1.02)	
Missing	1.54 (0.97-2.45)	
Widowed	0.99 (0.81-1.22)	
Gender: male vs female	1.00 (0.84-1.18)	.96
Age, y (reference, <55)		
55-64	1.40 (1.24-1.57)	<.001
≥65	1.45 (1.23-1.71)	
Education (reference, more than HS)		
HS GED or less	0.96 (0.87-1.06)	.02
Missing	1.53 (1.12-2.08)	
Employment (reference, employed)		
Unemployed	0.98 (0.86-1.10)	.86
Retired	1.01 (0.86-1.19)	
Missing	0.89 (0.62-1.28)	
Difficulty paying for basics (reference, no)		
Yes	1.00 (0.90-1.12)	.003
Missing	0.52 (0.35-0.75)	
Social support (reference, high or good)		
Low or poor	0.95 (0.86-1.05)	.07
Missing	1.55 (1.00-2.40)	
Emotional distress (reference, low)		
High	0.86 (0.77-0.96)	.01
Missing	0.70 (0.44-1.12)	
Self-reported health (reference, good or excellent)		
Poor or fair	0.86 (0.78-0.96)	.02
Missing	0.96 (0.75-1.23)	
Self-reported alcohol problem: yes vs no	0.90 (0.80-1.00)	.06
Self-reported drug problem: yes vs no	1.01 (0.88-1.18)	.85
Self-reported chronic pain: yes vs no	1.02 (0.92-1.13)	.72
Chronic homelessness: yes vs no	0.98 (0.87-1.11)	.75
Comorbidities		
Anxiety, yes vs no	0.81 (0.72-0.90)	<.001
Diabetes, yes vs no	1.22 (1.09-1.37)	.001
Traumatic brain injury, yes vs no	1.29 (1.00-1.65)	.05

Abbreviations: aOR, adjusted odds ratio; CBOC, community-based outpatient clinic; H-PACT, homeless patient aligned care team; HS, high school; PACT, patient aligned care team; VAMC, Veterans Affairs Medical Center.

^a^ Model controlled for all comorbidities listed in Table 1 (only significant results presented in Table 3), the station where the veteran was receiving primary care, and weighted by response likelihood.

^b^ *P* values are derived from the type 3 test and assess the overall effect of a factor in the model.

Unadjusted and adjusted comparisons of service utilization are shown in Table 4. In unadjusted analyses, those in H-PACTs had significantly more primary care visits (4.5; 95% CI, 4.4-4.6 vs 4.1; 95% CI, 3.9-4.2 visits), more homeless services visits (12.7; 95% CI, 12.2-13.2 vs 4.7; 95% CI, 4.1-5.4 visits), and fewer specialty care visits (6.0; 95% CI, 5.6-6.4 vs 8.3; 95% CI, 7.7-8.8 visits) than those in mainstream PACTs (each *P* < .001), with similar results after adjustment for potential confounders. In adjusted analyses, those in H-PACTs also had fewer ED visits (1.0; 95% CI, 0.9-1.1 vs 1.4; 95% CI, 1.3-1.5 visits; *P* < .001) than those in mainstream PACTs and were less likely to have any ED visits (OR, 0.83; 95% CI, 0.75-0.92).

**Table 4.** Comparisons of Service Utilization in the 12 Months Before Survey Completion Between Homeless Patient Aligned Care Team (H-PACT) and Mainstream PACT Clinics

Type of utilization	Population means (least square means) for service utilization, No. of visits (95% CI)					
	Unadjusted^a^			Adjusted^b^		
	H-PACT	Mainstream PACT	*P* value	H-PACT	Mainstream PACT	*P* value
Outpatient care						
Primary care^c^	4.5 (4.4-4.6)	4.1 (3.9-4.2)	<.001	4.6 (4.5-4.7)	4.0 (3.8-4.2)	<.001
Homeless services	12.7 (12.2-13.2)	4.7 (4.1-5.4)	<.001	12.0 (11.5-12.6)	5.8 (5.1-6.5)	<.001
Mental health	13.2 (12.2-14.2)	11.2 (9.9-12.5)	.02	12.1 (11.2-13.1)	13.0 (11.8-14.3)	.30
Specialty	6.0 (5.6-6.4)	8.3 (7.7-8.8)	<.001	6.2 (5.8-6.6)	7.9 (7.4-8.4)	<.001
Rehabilitation	2.9 (2.6-3.2)	3.6 (3.2-4.0)	.004	3.0 (2.7-3.2)	3.5 (3.2-3.9)	.02
Ancillary	3.6 (3.4-3.9)	4.7 (4.4-5.1)	<.001	3.7 (3.5-4.0)	4.6 (4.2-4.9)	<.001
Emergency department						
No. of visits^d^	1.3 (1.2-1.4)	1.5 (1.4-1.6)	.01	1.0 (0.9-1.1)	1.4 (1.3-1.5)	<.001
Any, relative risk (95% CI)^d^	0.86 (0.79-0.94)	1 [Reference]	.001	0.83 (0.75-0.92)	1 [Reference]	<.001
Inpatient care						
No. of admissions^d^	0.4 (0.38-0.47)	0.4 (0.36-0.46)	.58	0.3 (0.2-0.3)	0.3 (0.3-0.3)	.11
Any, relative risk (95% CI)^d^	1.00 (0.88-1.16)	1 [Reference]	.92	0.95 (0.82-1.11)	1 [Reference]	.56

^a^ Derived from regressions adjusted for station and weighted by response likelihood.

^b^ Derived from regressions adjusted for station and weighted by response likelihood as well as clinic type, facility type, race, ethnicity, marital status, gender, age, education, employment, difficulty paying for basics, social support, emotional distress, self-reported health, self-reported alcohol problem, self-reported drug problem, self-reported chronic pain, chronic homelessness, and comorbidities.

^c^ Primary care visits were limited to in-person as well as telehealth visits where a physician or advanced practice clinician was assigned as the primary practitioner for that visit; other types of visits were identified using Veterans Affairs stop codes without reference to the mode of delivery or practitioner type.

^d^ Negative binomial regression.

## Discussion

In this cohort study of linked survey responses and VA clinical data, we found that VHEs in H-PACTs had higher primary care continuity and more primary care visits without a rise in specialty visits or in ED use compared with mainstream PACTs. When considered alongside other studies showing better patient experience in H-PACTS,^11^ the findings provide further justification to the investment in tailored clinics for VHEs.

This study was designed to identify and scrutinize potential tradeoffs between primary care continuity and accessibility.^15^ For example, a team-based primary care model might aim to increase accessibility by employing advance practice clinicians to allow additional same-day appointments. While such activities could, arguably, provide for better actual continuity than an alternative model that shunted urgent visits to an urgent care center or ED, these additional visits could reduce the apparent measured primary care continuity (which depends on seeing the same clinician). However, it does not appear that continuity gains by H-PACTs in this study were at the expense of accessibility. Rather than finding evidence of substitution (ie, more ED use and more specialty care visits but potentially less primary care use), VHEs enrolled in HPACTs had higher continuity and more primary care visits without any corresponding increase in ED visits or specialty care utilization.

Previous studies have shown that tailoring primary care for vulnerable populations can improve health service utilization outcomes.^14^ For example, one study^36^ showed that veterans with substance use disorders and other social vulnerabilities had reduced ED use and hospitalization after enrollment in an integrated primary care clinic. To our knowledge, ours is the first study to show that tailored primary care for a vulnerable population is associated with higher primary care continuity. The finding is particularly important because the health care challenges resulting from lack of continuity in the care were animating concerns at the very inception of US-based Health Care for the Homeless Programs in the mid-1980s.^37^

Other published studies have generally found positive associations between higher continuity and health service utilization outcomes.^4,7,38^ However, there have been very few studies examining interventions or policy levers aimed at increasing continuity and what effect those would have on other outcomes.^39^ While increased continuity is desirable from both a patient and policy perspective, continuity is not necessarily an outcome that is amenable to direct manipulation through simple policies. Rather, increased continuity may be ideally conceptualized as a desirable byproduct of care delivery interventions. Therefore, continuity should be considered alongside measurement and comparison of more traditional outcomes of those interventions. In this case, the investment required for tailored clinics appears to improve the patient experience^11^ in addition to being associated with improved continuity.

Interestingly, we found that being enrolled in a clinic at a CBOC was strongly associated with high primary care continuity independent of whether the clinic was homeless-tailored or not. CBOCs are smaller and typically have fewer practitioners than their VAMC counterparts. Due to the way continuity is measured, it is not surprising that clinics with fewer practitioners might tend to have higher measured primary care continuity. However, patient populations also likely differ in important ways between CBOCs and VAMCs, and it was beyond the scope of this study to examine differences in care delivered at these various sites. Future studies should examine whether there are additional insights to be gleaned from differences in care delivery at these sites.

### Limitations

This study is subject to limitations. First, the study was cross-sectional in nature, where the visits used to calculate continuity occurred over the same time period as the other events being examined (eg, ED visits). While cross-sectional data limit the ability to draw causal inferences, additional care should be taken when interpreting these results; high continuity should be conceptualized as an exposure that has potential to improve subsequent care. Second, clinic assignment was determined at a single point in time during the observation period. It is possible that veterans changed clinics shortly before or shortly after this assignment was determined, so there could be misclassification, though any misclassification would likely bias the results toward the null. Third, we excluded survey participants who did not have enough primary care visits to permit a measure of continuity. Fundamentally, studies using measures of continuity necessitate regular engagement with the health care system. While there is every reason to be concerned about the care of vulnerable populations who do not regularly engage with primary care, our results are not generalizable to patients who do not regularly utilize primary care. Additionally, these data predate the COVID pandemic, and it is possible that care patterns have changed since that time.

## Conclusions

In conclusion, this study found that VHEs in H-PACT clinics had higher primary care continuity than those in mainstream PACT clinics, with no indication of substitution of specialty or emergency visits for primary care. Tailoring primary care for vulnerable populations appears to be associated with more efficient health care delivery, which could offset some of the costs in providing such care.

## Notes

Supplement 1. eTable 1. Stop Codes Used to Categorize Care Type for Encounters eTable 2. Baseline Participant Characteristics of 5766 Surveyed Veterans Experienced in Homelessness, Overall and by Number of Primary Care Visits in the 12 Months Before Survey

Supplement 2. Data Sharing Statement
