# Supplementary material for: Primary Care Continuity and Utilization Patterns for Veterans With Homeless Experience
Source: JAMA Netw Open. 2026 Feb 2;9(2):e2557754. doi: 10.1001/jamanetworkopen.2025.57754 (PMC12865654; doi:10.1001/jamanetworkopen.2025.57754)
Supplement: Supplement 1. — eTable 1. Stop Codes Used to Categorize Care Type for Encounters eTable 2. Baseline Participant Characteristics of 5766 Surveyed Veterans Experienced in Homelessness, Overall and by Number of Primary Care Visits in the 12 Months Before Survey [file jamanetwopen-e2557754-s001.pdf]

Supplemental Online Content

Riggs KR, DeRussy AJ, Hoge AE, et al. Primary care continuity and utilization patterns for homeless-experienced veterans. *JAMA Netw Open*. 2026;9(2):e2557754. doi:10.1001/jamanetworkopen.2025.57754

eTable 1. Stop Codes Used to Categorize Care Type for Encounters

eTable 2. Baseline Participant Characteristics of 5766 Surveyed Veterans Experienced in Homelessness, Overall and by Number of Primary Care Visits in the 12 Months Before Survey

This supplemental material has been provided by the authors to give readers additional information about their work.

© 2026 Riggs KR et al. *JAMA Network Open*.

eTable 1. Stop Codes Used to Categorize Care Type for Encounters

CareType	Stop Codes with Description
Primary Care	301 General Internal Medicine, 322 Comprehensive Women's Primary Care Clinic, 323 Primary Care Medicine, 338 Telephone Primary Care, 348 Primary Care Shared Appointment, 350 Geripact
Homeless Services	504 Grant & Per Diem - Group, 507 Hud/Vash - Group, 508 Hchv/Hcmi - Group, 511 Grant & Per Diem - Individual, 522 Department Of Housing And Urban Development (Hud)-Va Supported Housing (Vash) Individual, 528 Telephone Homeless Chronically Mentally Ill (Hcmi), 529 Hchv/Hcmi - Individual, 530 Telephone/Hud-Vash, 555 Homeless Veteran Community Employment Services - Individual, 556 Homeless Veteran Community Employment Services - Group,
Mental Health	156 Hbpc - Psychologist, 157 Hbpc - Psychiatrist, 292 Observation Psychiatry, 502 Mental Health Clinic - Individual, 509 Psychiatry, 510 Psychology, 513 Substance Use Disorder - Individual, 516 Ptsd - Group, 523 Opioid Treatment Program, 524 Active Duty Sexual Trauma, 525 Women's Stress Disorder Treatment Teams, 527 Telephone Mental Health, 533 Mental Health Intervention Biomedical Care - Individual, 534 Mental Health Integrated Care - Individual, 535 Mental Health Vocational Assistance - Individual, 536 Telephone Mental Health Vocational Assistance, 538 Psychological Testing, 539 Mental Health Integrated Care - Group, 542 Telephone/Post-Traumatic Stress Disorder (Ptsd), 545 Telephone/Substance Use Disorder, 546 Telephone Intensive Community Mental Health Recovery Services (Icmhr), 547 Intensive Substance Use Disorder - Group, 550 Mental Health Clinic - Group, 552 Intensive Community Mental Health Recovery Services (Icmhr)- Individual, 560 Substance Use Disorder - Group, 561 Pct-Post Traumatic Stress - Group, 562 Ptsd - Individual, 564 Mental Health Team Case Management, 565 Mental Health Intervention Biomedical Care - Group, 566 Mental Health Risk-Factor, Reduction Educational Group, 567 Intensive Community Mental Health Recovery Services (Icmhr)- Group, 568 Mental Health Compensated Work Therapy/ Supported Employment (Cwt/Se) , Face-To-Face, 573 Mental Health Incentive Therapy Face-To-Face, 574 Mental Health Compensated Work Therapy/Transitional Work Experience (Cwt/Twe) Face-To-Face, 575 Mental Health Vocational Assistance - Group, 576 Psychogeriatric Clinic - Individual, 577 Psychogeriatric Clinic - Group, 579 Telephone/Psychogeriatrics, 582 Psychosocial Rehabilitation Recovery Center (Prrc) - Individual, 583 Psychosocial Rehabilitation Recovery Center (Prrc)- Group, 584 Telephone Psychosocial Rehabilitation Recovery Center (Prrc), 586 Residential Rehabilitation Treatment Program (Rrtp) - Individual, 587 Residential Rehabilitation Treatment Program (Rrtp) - Group, 591 Incarcerated Veterans Re-Entry, 592 Veterans Justice Outreach, 593 Residential Rehabilitation Treatment Program (Rrtp) Outreach Services, 596 Residential Rehabilitation

© 2026 Riggs KR et al. *JAMA Network Open*.

	Treatment Program (Rrtp) Admission Screening Services, 597 Telephone/Residential Rehabilitation Treatment Program (Rrtp), 598 Residential Rehabilitation Treatment Program (Rrtp) Outpatient Individual, 599 Residential Rehabilitation Treatment Program (Rrtp) Outpatient Group
Specialty Care	**General:** 120 Health Screening, 290 Observation Medicine, 324 Telephone/Medicine, 331 Pre-Bed Care Md (Medical Service), 336 Medical Pre-Procedure Evaluation, 143 Sleep Study, 231 Cardio-Pulmonary Rehabilitation, 293 Observation Neurology, 302 Allergy Immunology, 303 Cardiology (Card), 304 Dermatology (Derm), 305 Endocrinology, 306 Diabetes, 307 Gastroenterology, 308 Hematology, 309 Hypertension, 310 Infectious Disease (Infx Dse), 311 Cardiac Implantable Electronic Devices (Cied), 312 Pulmonary/Chest, 313 Renal/Nephrology (Except Dialysis), 314 Rheumatology/Arthritis, 315 Neurology, 316 Oncology/Tumor, 317 Anti-Coagulation Clinic, 321 Gastrointestinal (Gi) Endoscopy, 325 Telephone/Neurology, 327 Medical (Med) Physician (Md) Perform Invasive Operating Room (Or) Procedure (Proc), 329 Medical Procedure Unit, 330 Chemotherapy Procedures Unit Medicine, 333 Cardiac Catheterization, 334 Cardiac Stress Test, 335 Padrecc (Parkinson's Disease), 337 Hepatology Clinic, 339 Obstetrics, 340 Genomic Care, 344 Multiple Sclerosis (Ms), 345 Epilepsy Center Of Excellence, 346 Als Center, 349 Sleep Medicine, 356 War Related Illness And Injury Study Center (Wriisc), 369 Electrophysiology Laboratory, 391 Cardiac Echo, 392 Ambulatory Ecg Monitoring, 394 Medical Specialty Shared Appointment, 420 Pain Clinic, 602 Assisted Hemodialysis, 603 Limited Self Care Hemodialysis, 604 Home/Self Hemodialysis Training, 606 Continuous Ambulatory Peritoneal Dialysis (CAPD), 607 Limited Self Care Continuous Ambulatory Peritoneal Dialysis (CAPD), 608 Home/Self Continuous Ambulatory Peritoneal Dialysis (CAPD) Training, 611 Telephone/Dialysis<br><br>**Geriatric/Extended:** 118 Home Treatment/Services, 119 Community Nursing Home (Cnh) Follow-Up, 121 Community Residential Care (Crc), 173 Hbpc - Social Worker, 174 Hbpc - Therapist, 175 Hbpc - Dietitian, 176 Hbpc - Clinical Pharmacist, 177 Hbpc - Other, 178 Telephone Hbpc, 190 Adult Day Health Care (Adhc) (Va-Based Adhc), 191 Community Adult Day Health Care (Adhc) Follow-Up, 319 Geriatric Evaluation And Management (Gem) Clinic, 326 Telephone/Geriatrics, 347 Als Home Care Program, 351 Hospice Care, 352 Grecc Clinical Demonstration, 353 Palliative Care, 354 Hospital In Home, 658 State Home Adult Day Health Care, 680 Home And Community-Based Care (HCBC) Assessment, 682 Va-Referrals To Hcbc Providers<br><br>**Surgery:** 291 Observation Surgery, 401 General Surgery, 402 Cardiac Surgery, 403 Otolaryngology (Ent), 404 Gynecology, 405 Hand Surgery, 406 Neurosurgery, 407 Ophthalmology, 408 Optometry, 409 Orthopedics/Joint Surgery, 410 Plastic Surgery, 411 Podiatry, 413 Thoracic Surgery, 414 Urology Clinic, 415 Vascular Surgery, 418 Amputation Clinic, 419 Anesthesia Consult, Including Pre-Procedure And Expanded

© 2026 Riggs KR et al. *JAMA Network Open*.

	Post-Procedure Assessment, 424 Telephone/Surgery, 427 Anesthesia Special Procedures In Operating Room Suite Or A Non-Or Procedure Room, 428 Telephone/Optometry, 429 Patient Care In Operating Room (Or), 430 Cysto Room In Urology Clinic, 432 Pre-Surgery Evaluation, 434 Non-Or Anesthesia Procedures, 435 Surgical Procedure Unit, 441 Telephone Anesthesia, 486 Cardiothoracic Surgery, 487 Bariatric Surgery, 488 Surgical Oncology, 489 Spinal Surgery, 718 Eye Telehealth Screening,
Rehabilitation Care	195 Polytrauma Transitional Rehabilitation Program Individual, 196 Polytrauma Transitional Rehabilitation Program Group, 197 Polytrauma/Traumatic Brain Injury (Tbi) Individual, 198 Polytrauma/Traumatic Brain Injury (Tbi) Group, 199 Telephone Polytrauma/Traumatic Brain Injury (Tbi), 201 Pm&Rs Physician, 202 Recreation Therapy Service, 203 Audiology, 204 Speech-Language Pathology, 205 Physical Therapy, 206 Occupational Therapy, 207 Pm&Rs Incentive Therapy Face-To-Face, 208 Pm&Rs Compensated Work Therapy/ Transitional Work Experience (Pm&Rs Cwt/Twe) Face-To-Face, 209 Vist Coordinator, 210 Spinal Cord Injury (Sci), 211 Pm&Rs Amputation Clinic, 213 Pm&Rs Vocational Assistance, 214 Kinesiotherapy (Kt), 215 Spinal Cord Injury (Sci) Home Care Program, 216 Telephone/Rehabilitation (Rehab) And Support, 217 Blind Rehab Outpatient Specialist (Bros), 218 Blind Rehab Center, 220 Visor & Advanced Blind Rehab, 221 Telephone Visual Impairment Service Team (Vist), 222 Pm&Rs Compensated Work Therapy/Supported Employment (Pm&Rs Cwt/Se) Face-To-Face, 224 Telephone Spinal Cord Injury (Sci), 225 Spinal Cord Injury (Sci) Telehealth And Virtual Care, 229 Telephone/Blind Rehab Program, 230 Pm&Rs Driver Training, 240 Physical Medicine And Rehabilitation Assistive Technology Clinic, 241 Wheelchair And Advanced Mobility Clinic, 250 Rehabilitation Services Group, 417 Prosthetics / Orthotics, 423 Prosthetic And Sensory Aids Service, 425 Telephone/Prosthetics/Orthotics, 437 Victors & Advanced Low Vision, 438 Intermediate Low Vision Care, 439 Low Vision Care
Emergency	130 Emergency Department
Ancillary	103 Telephone Triage, 123 Nutrition/Dietetics - Individual, 124 Nutrition/Dietetics - Group, 125 Social Work Service, 139 Health And Well-Being Services, 142 Wound Treatment & Ostomy Care, 147 Telephone/Ancillary, 159 Complementary And Integrative Health Treatment, 160 Clinical Pharmacy, 166 Chaplain Service - Individual, 167 Chaplain Service - Group, 169 Telephone/Chaplain, 180 Dental, 181 Telephone Dental, 182 Telephone Case Management, 328 Medical/Surgical Day Unit (Msdu), 332 Pre-Bed Care Rn (Medical Service), 372 Move! Program Individual, 373 Move! Program Group, 436 Chiropractic Care, 683 Home Telehealth (Ht) Non-Video Monitoring, 685 Care Of Home Telehealth (Ht) Program Patients, 686 Telephone Contact By Home Telehealth (Ht) Staff

© 2026 Riggs KR et al. *JAMA Network Open*.

[a] List of stop codes adapted from Ferguson 2021. Primary changes consist of a stricter definition of primary care and the creation of a Homeless Services Care Type (previously grouped with Mental Health)

Ferguson JM, Jacobs J, Yefimova M, Greene L, Heyworth L, Zulman DM. Virtual care expansion in the Veterans Health Administration during the COVID-19 pandemic: clinical services and patient characteristics associated with utilization. *Journal of the American Medical Informatics Association.* 2021;28(3):453-462. DOI: 10.1093/jamia/ocaa284.

© 2026 Riggs KR et al. *JAMA Network Open*.

eTable 2. Baseline Participant Characteristics of 5766 Surveyed Veterans Experienced in Homelessness, Overall and by Number of Primary Care Visits in the 12 Months Before Survey

© 2026 Riggs KR et al. *JAMA Network Open*.

	Overall<br><br>N = 5,766<br>(100.0%)	No Primary Care Visits<br>N= 885<br>(15.4%)	1 Primary Care Visit<br>N=983<br>(17.1%)	2+ Primary Care Visits<br>N=3,898<br>(67.6%)	P value
	N (%)	N (%)	N (%)	N (%)	
Primary Care Clinic Type					<.001
H-PACT	3,394 (58.86)	592 (66.89)	531 (54.02)	2,271 (58.26)	
Mainstream PACT	2,372 (41.14)	293 (33.11)	452 (45.98)	1,627 (41.74)	
PC Continuity					<.001
High (0.75 to 1.00)	3,404 (69.7*)	NA	983 (100.0)	2421 (62.1)	
Moderate (0.50 to <0.75)	1,125 (23.0*)	NA	0 (0.0)	1125 (28.9)	
Low (<0.50)	352 (7.2*)	NA	0 (0.0)	352 (9.0)	
Age, Mean (SD)					<0.001
<55	1,493 (25.89)	287 (32.43)	303 (30.82)	903 (23.17)	
55-64	2,977 (51.63)	453 (51.19)	493 (50.15)	2,031 (52.10)	
>65	1,296 (22.48)	145 (16.38)	187 (19.02)	964 (24.73)	
Gender					0.07
Female	506 (8.78)	67 (7.57)	74 (7.53)	365 (9.36)	
Male	5,260 (91.22)	818 (92.43)	909 (92.47)	3,533 (90.64)	
Race					0.16
White	2,598 (45.06)	377 (42.60)	431 (43.85)	1,790 (45.92)	
Black	2,402 (41.66)	387 (43.73)	409 (41.61)	1,606 (41.20)	
Other	698 (12.11)	107 (12.09)	136 (13.84)	455 (11.67)	
Missing	68 (1.18)	14 (1.58)	7 (0.71)	47 (1.21)	
Ethnicity					0.81
Hispanic	602 (10.44)	94 (10.62)	94 (9.56)	414 (10.62)	
Non-Hispanic	5,022 (87.10)	772 (87.23)	866 (88.10)	3,384 (86.81)	
Missing	142 (2.46)	19 (2.15)	23 (2.34)	100 (2.57)	
Marital Status					0.053
Married	1,058 (18.35)	140 (15.82)	194 (19.74)	724 (18.57)	
Never Married	1,522 (26.40)	268 (30.28)	270 (27.47)	984 (25.24)	
Divorced	2,691 (46.67)	402 (45.42)	448 (45.57)	1,841 (47.23)	
Widowed	384 (6.66)	58 (6.55)	54 (5.49)	272 (6.98)	
Missing	111 (1.93)	17 (1.92)	17 (1.73)	77 (1.98)	
Education					0.19
HS GED or equivalent	2,197 (38.10)	356 (40.23)	386 (39.27)	1,455 (37.33)	
More than HS GED	3,354 (58.17)	500 (56.50)	552 (56.15)	2,302 (59.06)	
Missing	215 (3.73)	29 (3.28)	45 (4.58)	141 (3.62)	
Employment					0.19
Employed	2,197 (38.10)	356 (40.23)	386 (39.27)	1,455 (37.33)	
Unemployed	3,354 (58.17)	500 (56.50)	552 (56.15)	2,302 (59.06)	
Retired	1,391 (24.12)	180 (20.34)	204 (20.75)	1,007 (25.83)	
Missing	163 (2.83)	31 (3.50)	32 (3.26)	100 (2.57)	
Facility Type					0.03
VAMC	4,270 (74.05)	625 (70.62)	724 (73.65)	2,921 (74.94)	
CBOC	1,496 (25.95)	260 (29.38)	259 (26.35)	977 (25.06)	

© 2026 Riggs KR et al. *JAMA Network Open*.

Chronically Homeless	1,033 (17.92)	198 (22.37)	166 (16.89)	669 (17.16)	<0.001
Elixhauser, Mean (SD)	3.9 (2.3)	3.1 (2.1)	3.3 (2.1)	4.2 (2.4)	<.001
Difficulty paying for basics					0.003
Yes	1,396 (24.21)	258 (29.15)	230 (23.40)	908 (23.29)	
No	4,236 (73.47)	602 (68.02)	730 (74.26)	2,904 (74.50)	
Missing	134 (2.32)	25 (2.82)	23 (2.34)	86 (2.21)	
Social Support					0.02
Low/Poor	2,135 (37.03)	371 (41.92)	349 (35.50)	1,415 (36.30)	
High/Good	3,536 (62.16)	497 (56.16)	616 (62.67)	2,423 (62.16)	
Missing	95 (1.65)	17 (1.92)	18 (1.83)	60 (1.54)	
Self-reported Health					<0.001
Poor/Fair	2,662 (46.17)	372 (42.03)	409 (41.61)	1,881 (48.26)	
Excellent/ Good	2,878 (49.91)	475 (53.67)	538 (54.73)	1,865 (47.85)	
Missing	226 (3.92)	38 (4.29)	36 (3.66)	152 (3.90)	
Self-reported Alcohol Problem					<0.001
Yes	1,624 (28.17)	299 (33.79)	300 (30.52)	1,025 (26.30)	
No	4,035 (72.14)	567 (64.07)	656 (66.73)	2,812 (72.14)	
Missing	107 (1.86)	19 (2.15)	27 (2.75)	61 (1.56)	
Self-reported Drug Problem					<0.001
Yes	782 (13.56)	143 (16.16)	157 (15.97)	482 (12.37)	
No	4,877 (86.07)	723 (81.69)	799 (81.28)	3,355 (86.07)	
Missing	107 (1.86)	19 (2.15)	27 (2.75)	61 (1.56)	
Self-reported Chronic Pain					<0.001
Yes	3,518 (61.01)	470 (53.11)	549 (55.85)	2,499 (64.11)	
No	2,248 (38.99)	415 (46.89)	434 (44.15)	1,399 (35.89)	

Abbreviations: CBOC, CBOC, Community-based Outpatient Clinic; GED, General Educational Development; HS, High School; H-PACT, Homeless Patient Aligned Care Team; PACT, Patient Aligned Care Team; VAMC, Veterans Affairs Medical Center

*Calculated amongst those with ≥1 PC visit

© 2026 Riggs KR et al. *JAMA Network Open*.
