# Supplementary material for: Primary Care Continuity and Utilization Patterns for Veterans With Homeless Experience
Source: JAMA Netw Open. 2026 Feb 2;9(2):e2557754. doi: 10.1001/jamanetworkopen.2025.57754 (PMC12865654; doi:10.1001/jamanetworkopen.2025.57754)
Supplement: Supplement 2. — Data Sharing Statement [file jamanetwopen-e2557754-s002.pdf]

Data Sharing Statement

Riggs. Primary Care Continuity and Utilization Patterns for Homeless-Experienced Veterans. *JAMA Netw Open*. Published February 02, 2026. doi:10.1001/jamanetworkopen.2025.57754

Data
Data available: No

Additional Information
Explanation for why data not available: It is very onerous to share VA data, even if it is de-identified
